# A decolonial feminist perspective on gender equality programming in the Global South

**DOI:** 10.1371/journal.pgph.0005556

**Published:** 2026-01-07

**Authors:** Ogochukwu Udenigwe, Judi Aubel, Seye Abimbola

**Affiliations:** 1 Faculty of Medicine and Health, School of Public Health, The University of Sydney, Sydney, New South Wales, Australia; 2 Grandmother Project–Change through Culture, Rome, Italy; 3 Grandmother Project–Change through Culture, Mbour, Senegal; Institute of Tropical Medicine: Instituut voor Tropische Geneeskunde, BELGIUM

## Abstract

The feminisms emerging from postcolonial regions of the Global South have critiqued gender equality initiatives that adapt to, rather than dismantle, racist, capitalist, and patriarchal systems. They call for an exploration of how these initiatives become hosts to oppressive forces that derail gender equality efforts and worsen the health and well-being of women and girls in the Global South. Drawing on decolonial feminist perspectives, this paper addresses two broad questions: How does a gender equality agenda rooted in capitalist structures impact the health and well-being of women and girls in the Global South? Have attempts at transforming gender equality in the Global South been solely liberatory? The authors examined constructions of gender equality in 17 program documents from contemporary women and girl-centred international NGO programs in the Global South. The findings revealed four key themes related to shaping gender equality in programs and their implications for the health and well-being of women and girls. These themes include: 1) Reinforcing hierarchical knowledge praxis (excluding or silencing knowledge originating from the Global South, 2) Culturalizing violence (violence as an intrinsic characteristic of culture in the Global South), 3) Labelling work as inherently liberating (depoliticizing poverty, glossing over exploitative economic practices), 4) Universalizing human rights discourses (emphasizing neoliberal assumptions of personhood). We conclude by recommending a delinking from Western narratives and instead integrating a decolonial feminist perspective into gender equality programs to uncover and dislodge the myriad manifestations of colonial influences. Failing to do so, international NGO programs and policies will either be counterproductive or limited to partial and temporary success.

## 1 Introduction

### 1.1 Women and girls: Frontiers for capitalist growth

Unbridled enthusiasm for improving and investing in the health and wellbeing of women and girls in the Global South ushered in an explosion of interventions from global governance institutions, corporations and NGOs for gender equality and health development. Several milestones in girl empowerment initiatives were established in the early twenty-first century including the formation of the United Nations (UN) Interagency Taskforce on Adolescent Girls, the World Bank’s Adolescent Girls Initiative, the inaugural plenary session on adolescent girls at the World Economic Forum, and the launch of Plan International’s Because I Am A Girl campaign and Nike Foundation’s Girl Effect [1]. The 2010s further advanced this agenda with the establishment of the first International Day of the Girl, the introduction of the UN Foundation’s Girl Up, and the UK Department for International Development’s Girl Summit. Gender equality, situated at the intersection of feminism and development, is integral to their agenda in the Global South, particularly in areas considered necessary for economic growth, such as health, education, and finance.

Transnational feminist collaborations have undoubtedly increased attention on women and girls’ issues, acknowledging that their experiences of oppression or privilege are shaped by socio-structural factors, including colonial and neocolonial influences, economic conditions, and global capitalism, all of which deepen gender disparities globally [2]. In light of this, the various feminisms emerging from postcolonial regions of the Global South have critiqued feminist aspirations and ideologies that adjust to, rather than dismantle, racist, capitalist, and patriarchal systems [3–5]. They argue that in failing to attend to the complex and fluid realities of contemporary women and girls, gender equality initiatives are becoming unsuspecting hosts to oppressive forces that derail gender equality efforts.

Feminist ideals of gender equality are increasingly reshaped within a market-driven framework. Development initiatives interpret the struggle for gender equality as a means of achieving economic returns, supported by strong and politically salient narratives of gender equality as “smart economics” [6]. Women and girls in the Global South are positioned as untapped resources for economic growth and perfect economic agents (entrepreneurs and/or consumers) who are capable of lifting their countries out of poverty. At the household level, they are conceived of as more rational and efficient economic actors compared to “irresponsible” men and boys [7]. As the “key to breaking poverty”, development initiatives place a greater responsibility on women to overcome poverty, with limited attention paid to global inequalities and injustices. They demand diversification of women’s roles, including household responsibilities and income generation responsibilities, without analogous expectations from men. However, feminizing responsibility in this manner, that is, by leveraging existing gender roles, only worsens the inequalities and vulnerabilities these efforts aim to tackle [8].

### 1.2 Women and girls: Victims and solutions

Interestingly, the narratives around capable women and girls from the Global South are overlaid with notions of oppressed, victimized and fully disempowered personhoods [9]. The irony of the contradiction in their dual role as both victims and solutions to development issues is not lost. Development initiatives have embraced this contradiction in the proliferation of discourses that centre on “global girl power” as a solution to achieving gender equality [10]. This girl power narrative relies on a combination of discursive constructions and activities that position women and girls in affluent societies as saviours of their counterparts in the Global South, mobilizing discourses that assume that gender equality has been achieved in the Global North [11]. Essentialist discourses portray all girls as industrious and altruistic; girls in the Global South are described as needing the right investment and intervention, often from the Global North, to help them delay motherhood, become economically active and provide for their families. Meanwhile, girls in the Global North, portrayed as rational, capable and amenable to the demands of the changing market, are able to produce social movements for change in the Global South if they participate in social media campaigns and purchase campaign merchandise [10].

The constructions are also visible in representations of sexualities. In the Global South, women and girls’ sexuality is pathologized, perceived as excessive and problematic, and a potential hindrance to their economic productivity, thus needing intervention. By contrast, women and girls in the Global North are depicted as agentic, sexually expressive, and self-commodified [11,12]. The girl power narrative, as a key driver of development, foregrounds economic rationality and individualism in constructing limited notions of girlhood. The complex histories, including colonialism, that have shaped the “poverty-stricken” versus “prosperous” portrayals of girlhood in the Global South and North, respectively, are obscured and rarely interrogated [10]. Consequently, such constructions, even when claiming to shed light on the realities of girls in the Global South, reproduce racist neocolonial tropes. Wilson’s critique of neoliberalism in population policies shows how these policies, even when framed around reproductive rights and choice, depict people in the Global South as hypersexual or sexually deviant [11]. These tropes significantly impact the health of women and girls by creating a hierarchical system in reproductive and maternal health support. The concept of stratified reproduction, originally coined by Shellee Colen, recognizes the dynamics of sexual politics and the political economy of reproduction, highlighting how support for reproductive and maternal health varies at the global level. Inherent in this concept is the idea that certain kinds of reproduction are encouraged and supported, as observed for the Global North, but stigmatized and discouraged for the Global South [11,13,14].

The preceding examples reveal the limits of a simplistic approach to gender equality. As Vergès [2] argues, a feminism that only fights for gender equality but refuses to confront systems of hierarchies, e.g., capitalist and colonial systems that produce marginalization, is ultimately complicit in marginalizing women. Similarly, Bell Hooks is determined to prevent feminism from becoming a movement that only cares about or reacts against male domination, without addressing other forms of group oppression related to gender, race, and class [15]. Doing so requires challenging fundamental ways of thinking about gender and how power and dominance are maintained within hierarchies. For example, a narrow and binary view of gender that focuses on women and girls as the main beneficiaries may successfully raise awareness for some, but it can also make non-binary, transgender, and gender-non-conforming people invisible within program frameworks.

Decolonial feminism, a term initially introduced by Lugones, represents a pluralistic and justice-oriented perspective on feminism [16–19]. It emphasizes the importance of acknowledging the legacies of colonialism and aims to dismantle the intersecting structures through which coloniality functions, including those related to gender, race, capitalism, and epistemological oppression. Decolonial feminist theories are resolute in restoring an anti-racist and anti-colonial stance to the fight for gender equality [5,16]. An anti-capitalist lens fundamentally examines and critiques the functioning, discourse, and values of capitalism, highlighting how they are naturalized via corporate culture and neoliberal ideology. This critique also extends to capitalist feminism, which emphasizes profit, competition, and accumulation, and upholds the belief that this corporate culture is the ideal for feminists globally [20]. An anti-racist critique uncovers and aims to dismantle the methods by which racist representations are preserved. It also challenges the suppression or hierarchization of racialized knowledge systems and ways of being and examines how these processes uphold epistemic and material injustices faced by communities historically marginalized through colonialism [5].

These lenses query strands of Western feminism that ingratiate themselves to the capitalist world, sexualize racialized bodies (men and women) and further victimize racialized women. A simplistic view of gender equality and the rights of women and girls undermines the links between capitalism and racism while ignoring the impact of colonial histories of the many countries of the Global South. The focus on women and girls’ issues as solely concerning male domination ignores the global, capitalist and colonial systems of power that endure today, and absolves the Global North of their responsibilities for racist policies directed towards the Global South that impact women and girls.

Decolonial feminists advocate for viewing gender not as a fixed category, but as a spectrum of lived experiences influenced by patriarchy, colonialism, and global capitalism. Like many colonial acts, the continued existence of gender binaries in development and NGO discussions is bound to colonial legacies that seek to erase or constrain people of the Global South [21]. Various decolonial scholars have argued that in precolonial contexts of many Indigenous and non-Western societies, not only did societies view gender as fluid and in multiple forms, but it was also understood in egalitarian terms, not in terms of subordination [21–23]. The heteronormative binary, as understood today, was forcibly imposed on these societies by European capitalism to reinforce gender as a hierarchy. Foregrounded in neoliberal ideals, gender was used to control bodies, labour, and reproduction. Efforts to reject gender diversity in the Global South aimed to erase their existence and replace them with a mirror image of the colonizers. Additionally, this binary continues to be employed to justify the criminalization of homosexuality, non-binary gender identities, and the decimation of kinship and family structures that did not align with the norms of the white bourgeois patriarchal family, including queer, decolonial, and Indigenous communities [21,24].

The fluid and context-dependent relationships between masculinities and femininities are rarely interrogated, thereby sustaining a rigid, mutually exclusive binary that casts men as inherently powerful and women as inherently powerless. This is evident in ways that development narratives emphasize “male responsibilities” and “women’s empowerment”. As Cornwall & Rivasour argue [25], development discourses predominantly operate within this binary framework with limited attention to nonbinary, queer, transgender, collective, or decolonial identities.

### 1.3 Paradoxes in NGO discourse and practice

Any discussion on interventions targeting women and girls in the Global South must consider the unique role of international NGOs. International NGOs are viewed as patrons of public interest and are regarded as representing the interests of the people, so much so that they have come to replace other forms of civil society organizations in many communities of the Global South, such as trade unions, welfare associations, and women’s organizations [26]. Their self-perception as benevolent, non-governmental, non-political, and non-ideological has been integral to constituting and reaffirming their humanitarian identity [27]. Banks et al. [28] caution against conceptualizing NGOs as a homogeneous sector, emphasizing that this could obscure significant differences in ideology, structure, and power relations. However, their continued prominence, shaped and sustained by dominant development discourses that portray NGOs as inherently people-centred and grassroots-oriented, necessitates a critical assessment of their performances against these standards.

The complex and paradoxical identity of NGOs has become the subject of intense debates. Having emerged in the Western world from models based on Eurocentric cultural principles, they have the tendency to align themselves with global agendas at national and sub-national levels in the Global South [27,29]. Given this, their modus operandi tends to disregard the worldviews and knowledge systems of the local communities where they serve, making them complicit in producing and reproducing problematic narratives of the Global South. Critical assessments have shown that while they have successfully incorporated altruistic values into their self-description, their practice and rhetoric are implicitly anchored on the political and economic relations of capitalist expansion [30]. Therefore, claims of addressing global inequality exist simultaneously with practices that privilege Western entrepreneurship and reproduce economic relations that uphold disparities between the Global North and South.

While international NGOs vary widely in their orientations and practices, various studies locate some of their operations within the philosophical premises of neoliberal and globalization paradigms through which hegemonic norms are transmitted, expressed and entrenched [27,30,31]. They rely on the logic of the corporate world with basic principles of economic reductionism, which positions market exchanges as solutions to social problems [26,32]. Practices and rhetoric arising from this logic are legitimized through Western knowledge systems that ultimately treat the “market” as a universal language of science. This has led to the purely technocratic agendas that dominate the NGO sector and favour positivist and quantitative methodologies for measuring impact.

The expansion of the capitalist system has relied on cultural transformations and the production of neoliberal citizens. These citizens are primarily encouraged to pursue private interests rather than actively engage in building a public sphere that ensures the welfare of the majority [30]. NGOs have become powerful tools in this transformation, as is evident through their underlying ideologies and premises. For example, their practices and discourse on empowerment and poverty alleviation in the Global South tend to privilege neoliberal notions of private gain through improved individual capacities to enter the market while rendering invisible the social and political causes of poverty, including inequitable Global North/South power relations [31]. Programs imply that with improved capacities, the responsibility for producing change in communities rests on individuals.

In line with the individualized, self-determining forms of neoliberal citizenship, projects function on the premise that if women and girls acquire particular kinds of knowledge and skills, they can take responsibility for solving systemic challenges, achieve social justice, and produce change for themselves and their community [7,33]. Such projects are rarely accompanied by wider socio-economic or cultural initiatives that could enhance the lives of women and girls through the inclusion of their community, support systems and other interest groups. The future of communities and societies is reduced to the symbol of a young girl while neglecting the essential structural and cultural resources they require to thrive [7,33]. Lemke notes that this rhetoric is a key feature of neoliberal governance, intended to render the social domain economic by reducing government services and shifting the responsibilities for social risks, such as ill health, unemployment, and poverty, to individuals [34]. This governance strategy is used to “control individuals without at the same time being responsible for them” [p201] because, by its logic, individuals have the responsibility to work to improve their own welfare with limited claims on the government.

The Eurocentric premise of the individual emphasizes detachment from religion, family, and community. Chatterjee argues that this perspective, which labels community attachment as a “relic of pre-modern tradition” [35], is evident in the normative discourses of NGOs that portray these community forms as conservative, intolerant, and obstacles to the health and well-being of women and girls. Groups that could promote girls’ voices and interests are often overlooked, even when they can offer the most effective support systems. Through their networks, girls gain access to authorities who possess significant power and can drive changes that benefit them.

### 1.4 Arguments of the paper

The preceding arguments necessitate an exploration of how narratives on gender equality, and by extension, the health and well-being of women and girls in the Global South, are intertwined with frontiers of capitalist growth. While the authors recognize that efforts for gender equality involve various partnerships with development organizations, such as international NGOs, bilateral and multilateral agencies, and universities, we focus on international NGOs because of their perception as the preeminent organizational form capable of implementing global commitments through grassroots approaches within local communities [30]. Adopting a decolonial feminist perspective, this paper scrutinizes the role of NGOs in creating and perpetuating problematic narratives about the Global South that exacerbate the inequalities and vulnerabilities faced by women and girls, all under the guise of promoting gender equality.

The authors would like to preface this work by saying that while we do not doubt the good intentions of NGOs, the goal is to analyze the objective effect of actions regardless of intentions. We acknowledge the inherent tension and structural challenges NGOs face when fully disavowing the issues discussed. Although these organizations may criticize the capitalist and neoliberal frameworks that further marginalize women and girls, their ability to act independently can be limited by their reliance on financial support from donors whose operations are embedded in these same frameworks. As they navigate and continually negotiate the limits of donor priorities and funding constraints in their pursuit of transformative change, they may employ complex strategies in framing their advocacy, programming, and language [6,28]. Throughout the paper, we use the highly contested terms ‘Global South’ to denote regions of the world targeted for development, and ‘Global North’ to indicate the regions that initiate and fund interventions. The debates over the usage of these terms are not resolved in this paper; however, we draw inspiration from Teixeira’s [36] decolonial theorizing, which reclaims the term “Global South” to recognize shared colonial histories and Mignolo (2009) assert epistemic disobedience [37] where thinkers and movements in the Global South are actively engaged in the “messy work” of delinking from ossified Eurocentric ways of knowing and are themselves refusing to be silenced as producers of knowledge, culture and science.

## 2 Methods

Against the backdrop of the political economy of gender equality programs by international NGOs and their contradictory, universalizing, and essentialist discourses, this paper attends to decolonial feminist Abu-Lughod’s call to question the “empowerment” agenda for women and girls in the Global South, who are often portrayed as victims of culture and traditions. This call served as the basis for this paper to address the following questions: What are the implications of a gender equality agenda that has its roots located in the dynamics of capitalist structures? Have attempts at transforming gender equality in the Global South been purely liberatory?

In addressing these questions, the authors conducted document reviews to explore constructions of gender equality in contemporary women and girl-centred international NGO programs in the Global South. We adapted the READ approach described by Dalglish et al., [38] for collecting documents and gaining information in a health policy context at any level. The READ approach involves a systematic procedure for gaining information from documents, comprising **R**eadying materials, **E**xtracting relevant data, **A**nalyzing the data, and **D**istilling the findings.

1. Readying materials (document search and collection): Documents for review were collected between April and August 2024. The sampling for documents was purposeful; we searched for documents that described NGO programs and policies, specifically women and girl-centred programs that directly addressed gender inequality in countries of the Global South. We were interested in manifestations of gender equality so as to elaborate on its constructs and variations within women and girl-centred programmes. Documents used in this analysis included grey literature obtained from organizational websites, such as training documents, program plans, guidelines, and reports produced outside of formal publication channels. Key search terms were gender equality, women, and girl-centred. While there was no time limit on the search for documents, we consulted documents produced within the past 10 years to reflect current programming. We were interested in variations but shared patterns across organizations, that is, how the concept is interpreted and operationalized in diverse ways with the goal of building a nuanced understanding of the construct and its application across settings. To ensure relevance to contemporary programming, we limited our selection to documents published within the past 10 years. To maintain diversity while avoiding overrepresentation by any single organization, we capped the number of documents per organization at 2. Selection was further constrained by the electronic availability and accessibility of the documents. We focused on identifying common themes or goals on promoting gender equality that may manifest differently depending on the organizational or cultural setting.
2. Extracting relevant data: We consulted 17 documents related to women- and girl-centred programs and performed close readings, extracting information on authorship, summaries and key arguments of the documents, mentions of international or regional recommendations (See Table 1). A pre-determined extraction table was used as a guide to begin, and parameters were re-adjusted during extraction as needed. For example, information on mentions of international or regional human rights instruments/recommendations was added during data extraction. OU extracted the data. To enhance the credibility of the coding process, discrepancies/conflicts were resolved through periodic discussion with SA and JA, who are well-versed in qualitative and decolonial frameworks. These discussions also covered emerging codes and themes. We have included sample excerpts in (S1 Table: Code Book and Selected Supporting Quotes from Documents) to illustrate the traceability from raw text to code and overarching themes.
3. Analyzing the data: A qualitative approach was used to analyze the extracted data using thematic analysis [39,40]. Data were coded based on theoretical categories extracted from the literature on decolonial feminist analysis of gender equality programs. These categories included emergent codes that underscore contemporary notions of gender equality. Emerging themes were developed from the various codes. We analyzed symbolic gestures of addressing gender issues within the rhetoric of gender equality enmeshed in neoliberal contexts and established links between NGO practices and their coloniality of knowledge. Although our main analysis centers on hierarchies formed through North-South interactions and their influence on program modalities, we recognize that these modalities can intersect with social differences like class, caste, sexuality, ethnicity, migration status, and more, resulting in diverse experiences.
4. Distilling the findings: The data are presented in tables and text.

**Table 1:** Summary of documents.

	Document Name	Date	Author(s)	Document Type	Summary	Key Points Listed in Documents	Instruments Consulted
**1**	**BMGF Youth Empowerment Model**	2015	Bill & Melinda Gates Foundation, KIT	Conceptual Model	Conceptual model for empowering women and girls by transforming power dynamics, enabling voice and choice.	Defines empowerment as expansion of voice and choice Stresses systemic change and social transformation Prioritizes measuring empowerment outcomes Includes intersectional analysis of inequalities	Human rights framework
**2**	**Building Assets Toolkit**	2015	Judith Bruce, Sarah Engebretsen, Kimberly Glazer (Population Council)	Tool Kit	Guide for designing girl-centered programs using an asset-building approach tailored to local realities and aspirations.	Defines assets (human, social, economic, cognitive) Focuses on measurable, positive benchmarks Emphasizes age-appropriate, lifecycle programming Promotes community engagement and data-driven design	None explicitly stated
**3**	**ENRICH COVID-19 Impact Report**	2021	World Vision Canada	Report	Evaluates how Adolescent Girl Power Groups (AGPGs) in Bangladesh adapted during COVID-19.	AGPGs built resilience, confidence, and agency Promoted gender equality and SRHR awareness Continued engagement during lockdown Mitigated harmful practices and strengthened mental health coping	None explicitly stated
**4**	**Every Last Girl**	2016	Save the Children UK & International (multiple authors)	Report	Advocates for girls’ rights to education, protection, and voice; identifies major barriers and proposes systemic solutions.	Focus on child marriage, lack of services, lack of voice Girls’ Opportunity Index ranks country risks Calls for fair finance, equal treatment, and accountability Promotes data disaggregation and cross-sector investment	UN convention on the rights of a child + African Common Position on the AU Campaign to End Child Marriage
**5**	**FGM Technical Guidance 2022**	2022	UNFPA, UNICEF, WHO, UN Women, OHCHR	Report	This technical note provides comprehensive guidance to accelerate efforts to eliminate female genital mutilation (FGM), with a focus on human rights, prevention, services, and accountability.	FGM is a human rights violation- Prevention and community engagement are critical Multi-sectoral coordination is essential Health, legal, and social services must be integrated National laws and accountability mechanisms should be strengthened.	Various Inernational and regional frameworks: for example: Universal Declaration of Human rights + Protocol to the African Charter on Human and prople’s Rights on the Rights of Women in Africao (The Maputo Protocol)
**6**	**Funding for Empowerment of Girls: FGM Programming**	2020	UNFPA, UNICEF Joint Programme on FGM	Report	The document outlines the financial contributions and gaps in funding for girl-centered FGM elimination programming, highlighting donor contributions and recommendations for better funding strategies.	Importance of sustained funding for FGM programmes Emphasis on empowering girls as central to ending FGM- Donor commitments and funding gaps identified Accountability and transparency in resource allocation are necessary Recommends more donor engagement and flexible financing.	New York Declaration on Refugees and Migrants
**7**	**Strong Girls Make Strong Women: A Handbook for Girls’ Clubs**	2018	Julia Fan, Susan M. Blaustein (WomenStrong International)	Curriculum	A practical guide for creating and running girls’ clubs with a curriculum aimed at empowering girls ages 10–14 with life skills, leadership, health education, and community participation.	Emphasizes localized, adaptable content; promotes girls’ leadership, rights awareness, financial literacy, and life skills; designed for ease of implementation in low-resource settings.	Universal Declaration of Human Rights + Declaration on the Rights of the Child
**8**	**Gender-Transformative Collective Action: 7-Step Guide**	2023	Girls Not Brides, with UNFPA/UNICEF contributions	Guide	A facilitation guide for CSOs to engage in collective gender-transformative actions addressing child marriage and systemic gender inequality.	Steps include readiness, self-assessment, intensive workshops, roadmap creation, pilot programs, and reflection; targets root causes of gender inequality; emphasizes collective strategy and localized adaptation.	None explicitly stated
**9**	**Girl-Centered Program Design Toolkit**	2010	Karen Austrian & Dennitah Ghati (Population Council)	Toolkit	Toolkit to help organizations develop effective, safe, and targeted programs for adolescent girls, especially vulnerable ones, across diverse settings.	Focus areas: needs assessments, safe space creation, economic empowerment, SRHR, GBV response, leadership, and family/community engagement; supports data-driven program structuring.	None explicitly stated
**10**	**Girls Decide – Part 1: Programme Guidance**	2023	Melinda van Zyl, Clare Feinstein, Claire O’Kane, Luisa Dietrich	Programme Guide	Programme design guidance for life skills interventions targeting displaced or migrant girls to enhance resilience, ederment, and rights awareness.	Integrates girl-centered planning, participatory feedback, safeguarding, and adaptation to age, context, and culture; promotes social protection, inclusion, and contextualized delivery.	Emphasis on child rights but no instrument explicitly stated
**11**	**IDG 10-Year Report – Realising Every Girl’s Right to Flourish**	2022	Patricia Martin & Andre Wiesner. Plan International	Report	A review of global progress in girls’ rights on the 10th anniversary of the International Day of the Girl, evaluating changes from 2012–2022 across key areas like education, SRHR, and participation.	Identifies systemic progress and persistent gaps; calls for strengthened legal implementation, data systems, and girl-led policymaking; contextualized by global crises and local barriers.	Convention on the Elimination of All Forms of Discrimination Against Women + United Nations’ Convention on the Rights of the Child
**12**	**What Works for Adolescents’ Empowerment: A Learning Review**	2020	ICRW (Nanda et al.)	Report	A synthesis of programmatic lessons from adolescent empowerment initiatives in India over a decade, emphasizing what strategies are effective and scalable.	Successful strategies include convergent programming, addressing masculinities, creating safe spaces, building agency, promoting sports and aspirations, and tackling everyday violence; offers practical insights for replication and scale.	Adheres to the mandates of the Office of the United Nations High Commissioner for Human Rights + Prohibition of Child Marriage Act, India
**13**	**From Advocacy to Action**	2023	Khurana et al., ICRW	Report	Examines girl- and youth-led systems accountability in India, Kenya, and Uganda through participatory case studies and lessons from grassroots advocacy.	Youth-led monitoring, participatory budgeting, girl-led advocacy, and systems strengthening were critical to improving accountability and services for adolescents.	United Nations’ Convention on the Rights of the Child + African Charter on the Rights and Welfare of the Child + United Nations Human Rights Declaration
**14**	**Let Girls Lead Curriculum**	2014	Grace Kaimila Kanjo, Eugenia López Uribe, et al.	Curriculum	A comprehensive curriculum on girl-centered advocacy promoting rights, leadership, and systems change through storytelling, research, and peer-led strategies.	Introduces advocacy cycles, political mapping, proposal writing, alliance building, and self-care for girl leaders; highlights impact in Malawi, Liberia, Guatemala.	Declaration of Human Rights + The Convention on the Rights of the Child
**15**	**Partnering to Realize the Girl Effect**	2018	Nike Foundation and NoVo Foundation	Report	A decade-long evaluation of the Girl Effect strategy showing how investing in adolescent girls catalyzes societal transformation.	Safe spaces, economic empowerment, systemic reform, and leveraging girl champions are effective; partnerships and innovation central to scaling.	Global agendas (such as SDGS)
**16**	**Out of Time – Real Choices, Real Lives**	2024	Plan International (Dr. Kit Catterson et al.)	Report	Explores the impact of unpaid care work on girls’ time use and aspirations across nine countries through longitudinal qualitative research.	Girls face unequal time burdens; unpaid care limits education, rest, and leadership; early socialization into gendered roles reinforced by poverty and norms.	Convention of the Rights of the Child + African Charter on Children’s Rights
**17**	**Yes I Do Baseline Synthesis**	2018	Yes I Do Alliance (Plan, Rutgers, etc.)	Report	Baseline analysis of child marriage, FGM/C, and teenage pregnancy across 7 countries to guide context-specific interventions.	Community engagement, youth participation, SRHR education, legal reform, and alternative pathways like education and livelihoods are essential to prevent harmful practices.	None explicitly stated

### 2.1 Positionality of the authors

OU is a global health feminist researcher with a deep interest in how colonial legacies influence health and development practices. As a Black Nigerian woman trained in the Global North, my personal and professional background shape the questions I ask and the perspectives I bring to data interpretation. I acknowledge that my positionality, shaped by education, mobility, and linguistic access, affords me authority within global health spaces that remain inaccessible to many who are subjects of research. By writing on this topic, I leverage my positional privilege and institutional access to elevate scholarship from the margins, centre underheard voices, and interrogate universal, Western-centric assumptions that define womanhood and girlhood in global health. My engagement with decolonial feminist scholarship contributes to knowledge generation that remains accountable to the diverse social, cultural, and political contexts from which it originates.

JA is an American public health anthropologist with more than 25 years of experience in Senegal. Her work is rooted in a deep respect for local contexts, culture, and knowledge systems. As a researcher and practitioner, she has focused on fostering genuine collaboration and highlighting the everyday realities of the communities she serves. She co-founded and serves as the executive director of the Grandmother Project - Change through Culture, an organization challenging top-down, and Eurocentric, development strategies by valuing the wisdom and leadership of grandmothers and other local elders, whose knowledge and influence are often overlooked in mainstream approaches. She played a leading role in developing the Girls’ Holistic Development program that seeks to address issues like girls’ education, child marriage, teen pregnancy, and female genital mutilation. The program prioritizes the voices of Senegalese girls and their families, ensuring that interventions align with their values, aspirations, and lived experiences.

SA: I study how knowledge practices in health systems are shaped by colonial legacies and how to undo those legacies. As a Nigerian man trained primarily in Nigeria now working in Australia, my personal and professional background shape my work as a scholar, including the approach to data analysis and interpretation in this paper. By design, the epistemic infrastructure of the field (public health) and institution (academia) in which I work, silence and disregard the knowledge or marginalised others, whether in the Global South or the Global North. I take it as a primary responsibility to resist the ever-present tendency to reproduce the colonial logics that I seek to interrogate and undo in my work. This responsibility occasioned by the privilege that allowed and is afforded by my presence in academia as a scholar. As a result, I work to use academia as platform to transform entrenched knowledge practices that violate the dignity of marginalised people as knowers. This preoccupation is reflected in the work presented in this paper.

## 3 Findings and discussion

In this section, we examine four key themes related to constructing gender equality in programs focused on women and girls: establishing hierarchical knowledge praxis, culturalizing violence, labelling work as inherently liberating, and universalizing human rights discourses. Please see Table 2 for how these theses appear across the 17 documents reviewed for this analysis.

**Table 2:** Themes identified across documents.

	Organization	Report Title	Hierarchical Knowledge Praxis	Culturalizing Violence	The Vision of Work as Intrinsically Liberating	Universal Human Rights Discourses in Local Settings
**1**	**Bill and Melinda Gates**	**BMGF Youth Empowerment Model**		Violence is positioned as being enacted by communities and rooted in unequal gender norms.	Empowerment is emphasized in economic terms, income-generating opportunities are important, but it may not in and of itself, unlock opportunities for women and girls as the report emphasises	Emphasis on the individual in terms of human rights. Care work is generally constructed as a hindrance to the lives of women and girls.
**2**	**Population Council**	**Building Assets Toolkit**	Communities are conceptualized as lacking the capacity for women and girls’ organizing. Existing women and girl-only spaces to enhance health-related discussions are not sufficiently acknowledged or leveraged.	Culture is blamed for continuing violence inflicted on women and girls. Marriage systems that differ from those recognized in Western societies are portrayed as illegal		
**3**	**Save the Children**	**Every Last Girl**	Programs seem to have limited engagement with communities as knowledge holders. Important to centre community knowledge systems and resources in protection of women and girls	Customary laws and traditions are portrayed as inherently problematic for addressing gender inequality.	Programs promote the idea that employment is inherently liberating and will tackle gender inequality.	Emphasis on the universality of international instruments to address health rights of women and girls.
**4**	**World Vision**	**Adolescent Girl Power Groups:** **Building Resilience**	Shared histories of women’s power through organizing for health-related matters in women and girls-only spaces are essentially muted; instead, programs make claims to the newness of these concepts within adolescent girls’ power groups.	Program makes the claim that the only urgent threats to the safety of women and girls within their communities are deeply engraved traditional practices		
**5**	**UNFPA, UNICEF, WHO, UN Women, OHCHR**	**FGM Technical Guidance 2022**	The need to create safe spaces is emphasized in the program, but existing safe space structures are not explored.		Conceptualizes economic opportunities as the paramount metric for empowerment but overlooks important social considerations	Program challenges FGM/C, but foregrounds solutions to the practice through interpretations of human rights exclusively as individual rights.
**6**	**UNFPA, UNICEF Joint Programme on FGM**	**Funding for gender equality and the empowerment of women and girls in humanitarian programming**	Recognises women’s leadership in humanitarian situations, not as passive victims and aid beneficiaries, but as front-line workers			
**7**	**WomenStrong International**	**Strong Girls Make Strong Women: A Handbook for Girls’ Clubs**		Culture, amongst other things, undermines girls’ rights Many forms of gender-based violence are rooted in customs and culture		Communities limit girls from enjoying human rights under law
**8**	**Girls not Brides**	**Gender-Transformative Collective Action: 7-Step Guide**	Claim that traditionally, safe spaces are used to teach girls a limited set of gender-stereotyped productive skills.			
**9**	**Population Council**	**Girl-Centered Program Design Toolkit**		Program acknowledges other forms of violence impacting women including politically motivted violence, workplace related		Individualistic framing of human rights
**10**	**Save the Children**	**Girls Decide**	Preconceptions of program are that safe spaces or womentorship do not exist for women and girls.	Program focuses on traditional norms as the major cause of violence towards women and girls	Emphasizes women and girls’ access to job market as intrinsically linked to self- protection and regulation, particularly in the face of global issues such as displacements and decision-making	
**11**	**Plan International**	**IDG 10-Year Report – Realising Every Girl’s Right to Flourish**				Cultural values positioned as anti-rights. Recommendations to achieving lasting gender equality focuses on legislature to ensure the rights of women and girls
**12**	**ICRW**	**What Works for Adolescents’ Empowerment: A Learning Review**	Some programs admit to pre-existing organic forms of collective organizing; however, most focused on creating spaces with limited discussion on consulting or leveraging existing forms of women’s leadership		Programs focus on paid work as key to delaying marriage, increasing autonomy, and ensuring health and well-being. A flaw with this approach is that work is viewed as an automatic guarantee of freedom, and it accepts without question the unjust systems and institutions of society, especially capitalism.	
**13**	**ICRW**	**From Advocacy to Action**		Cultural norms are presented as the primary challenge to the health and well-being of women and girls. Violence is viewed as stemming from patriarchy, with limited exploration of other forms of structural violence meted out to women and girls		
**14**	**Let Girls Lead**	**Let Girls Lead Curriculum**	Potentials solutions to issues faced by women and girls seem to lie outside of the family and community; therefore, there was limited emphasis on leveraging already existing support systems for girls	Program makes the claim that female children are despised due to deeply rooted cultural beliefs and practices and are therefore most vulnerable to violence		The program recognised traditional authorities as part of legal systems and plan to use leverage their influence in reforms for women and girls’ health.
**15**	**Nike Foundation and NoVo Foundation**	**Partnering to Realize the Girl Effect**	Safe spaces are positioned as an innovative community-based or informal model of engaging women and girls		With claims that the worst possible outcomes for girls are poverty and unwanted pregnancies, the program lauds the potential for the private sector to expand economic opportunities for women and girls, with no mention of the quality of economic opportunities	The concept of human rights is primarily rooted in Western liberal individualism, with insufficient attention given to the significance of group rights.
**16**	**Plan International**	**Out of Time – Real Choices, Real Lives**			Program highlights the importance of care work to society and its advantages for women, men, and children. They point out the necessity for communities, governments, and NGOs to acknowledge their role in redistributing the burden of unpaid care work, as well as the importance of including the perspectives of girls and women in policies and decisions that affect their lives and how they allocate their time.	
**17**	**Yes I do Alliance**	**Yes I Do Baseline Synthesis**	Insufficient recognition of women’s positions of power and co-creators in addressing FGM which underscores the need to depatriarchize understandings of FGM to promote women’s health	Threats to women’s and girls’ health posed as cultural/traditional		Program challenges FGM/C and child marriages, but foregrounds solutions to the practice through interpretations of human rights exclusively as individual rights.

### 3.1 Hierarchical knowledge Praxis

This theme manifests especially in relation to how the concept of “safe spaces” is framed and deployed in international NGO programming. The concept of “safe spaces” is widely adopted and has arguably become an “overused but undertheorized’ metaphor in girl-centered projects in the Global South [41]. Their history as forms and sites of collective engagement where women (specifically African women) negotiated their complex political, social and cultural environments are rarely acknowledged. On the other hand, decolonial discourse continues to challenge such direct and indirect manifestations of colonial dominance and global hierarchies in knowledge praxis. By refocusing our attention on the systems that uphold knowledge production and how reality and truth are constructed, these discourses reject epistemic hierarchies that favour Western knowledge at the expense of non-Western knowledge systems [42,43]. Many have sought to unveil lost histories and knowledge systems that were deliberately excluded, suppressed, and erased [19].

Feminist authors Gumbonzvanda et al., (2021) decolonize the safe spaces knowledge base by positioning the concept, more broadly, as a tactic originating within African cultural histories where women have leveraged culture, emotions and narrative to shift harmful social norms, enhance empowering dialogue between girls and young women, and enable healing and counselling support, all to improve the health of women and girls [41]. Networks of women have long gathered on sacred grounds in quiet resistance or to vigorously represent their interests [44]. For centuries, the Shona/Bantu ethnic groups designated spaces called Nhanga (meaning girls’ room) as sites for initiating girls into adulthood. Here, girls discussed health issues, sexuality, marital conduct, as well as virtues of a moral life [41]. Similarly, in the Igbo ethnic group of Eastern Nigeria, traditional women’s networks gathered on sacred spaces (ilo mgbeleme) to address social, economic, political, and spiritual issues impacting women’s health and well-being. Bound by obligations to be “their sister’s keepers,” these women, regardless of social status and class, formed networks of organization whose dynamics tended toward the promotion of shared communitarian values rather than divisive individualistic values [44]. Historically, these forms of organizing steered women through politically turbulent times, such as the women’s resistance to colonial policies in the late 1920s and early 1930s.

Safe spaces as historical forms of collective engagement have been pivotal in conceptualizing Global South women’s organizing and achievements in the context of their everyday struggles. While commonly referred to in development work as originating within second-wave feminist praxis in the Global North, safe spaces have pre-colonial histories as more than simply a response to a static and predeﬁned category of “unsafe,” but have long been sites for intergenerational womentorship, health and sex education, social intelligence, and feminist organising [41].

Yet, girl-centric programs such as Nike Foundation make reference to the creation of safe spaces in their description of innovative community-based informal models. Similarly, programs such as the Population Council and World Vision, which define safe spaces as places where women and girls go to make connections and learn from female role models, often claim to have “created” such spaces in the Global South, suggesting a claim to the newness of this concept [45–47]. There is little acknowledgment of existing safe space structures. When programs such as Girls Not Brides do, in fact, acknowledge the history and existence of such spaces in African contexts, they tend to misrepresent their primary purpose by claiming they exist solely to teach girls a limited set of gender-stereotyped productive skills [48].

Central to a decolonial feminist argument is the assertion that such practices denying the Global South populations as knowledgeable and knowledge-producing are linked to ongoing modes of coloniality that continue to ignore, exclude, silence, and render invisible the knowledge originating from the Global South [16,19,49–51]. When such erasures shape foundational assumptions that inform development work aimed at women and girls, their diverse forms of leadership are delegitimized, thereby perpetuating the singular, monolithic imagery of the powerless and patriarchally oppressed Global South woman/girl [52].

Additionally, reclaiming traditional innovation is necessary to challenge and problematize the normative modes of thinking that continue to reflect coloniality in preconceptions of directionality of knowledge and learning. Terminology such as “reverse innovation,” referring to ideas adopted by the Global North from the Global South, superficially indicates the bidirectionality of knowledge forms [53,54]. However, this terminology is steeped in a colonial worldview that positions the Global North at the centre of innovation and the movement of knowledge, suggesting it is exceptional, “reverse”, when knowledge moves in the other direction. Such notions undervalue the histories of innovation emerging from the Global South and reinforce the hierarchy of forms of knowledge.

### 3.2 Culturalizing violence

This theme manifests in how international NGOs framed and deployed narratives on gender-based violence within Global South contexts. All the selected girl-centered programs acknowledge the pervasiveness of gender-based violence (GBV) and cite it as a barrier to women’s health and well-being. They recognized that while considerable progress has been made in addressing GBV, particularly through the influence of feminist research, much needed to be done. However, there was heavy emphasis on traditional practices as women and girl’s prime oppressors. Culture, as many reports argue, constitutes a key driver of GBV because women and girls’ vulnerabilities are deeply rooted in and exacerbated by traditional practices [55–59]. The Let Girls Lead program, for example, claims that female children are despised in Malawi due to deeply rooted cultural beliefs and practices and are therefore most vulnerable to violence [58]; and Save the Children singles out culture as a justification for violence and abuse [59]. The salient narrative on gender-based violence seems to be that since women and girls [in the Global South] live in patriarchal societies that are characterized by unequal gender power relations, they are at much greater risk for gender-based violence due to deeply entrenched socio-cultural norms. All will be well if we engage men and boys [often the perpetrators of violence] to recognize issues of masculinity and change their behaviour towards women and girls.

Undoubtedly, violence against women is a serious issue worldwide that must be addressed. In tackling culture head-on, Disele-Pitso reflects on how culture in postcolonial states has been co-opted and weaponized as a means to control and silence women (of which violence has been a means) [60]. Indeed, Razack agrees that effective anti-violence strategies must account for culture-specific ways that violence is enacted against women [61]. However, the dangers of culturalizing violence, that is, viewing it as an intrinsic characteristic of culture and attributing it solely to patriarchal societies in the Global South, is that it oversimplifies women and girls’ experiences within the global state of violence and falls headlong into producing racist ideologies of cultures of the Global South [61,62].

Cultures of the Global South (and racialized people in general) are portrayed as exceptionally violent towards women, in contrast to modern, Western cultures dominated by White people, obscuring the fact that White women also experience violence from White men. This has the effect of positioning White men as superior and non-patriarchal compared to men from the Global South. This “outsourced” patriarchy can explain why acts of violence in the Global South are deemed “cultural,” while violence in the West is blamed on individual behaviour or simply referred to as “violence,” not culture [49,61].

Decolonial theorizing explains how the colonial encounter contributed to the emergence and perpetuation of patriarchy. Colonialism undermined traditional values that protected against violence towards women, restructured societies, and shaped norms that reinforced hierarchies between men and women. Oyewumi discusses the role of colonialism in creating gender as a hierarchical system of power among the Yoruba people of Nigeria [23]. This structural bias, Nzegwu notes, was further entrenched in colonial social and economic policies that relegated women to the status of dependent minors, stripping them of their historical power [44,63]. Manning confirms a similar pattern, linking the patriarchy ingrained in Christianity, which was imposed on the Maya people of Guatemala during colonialism, to the emergence of a machismo society that confined women to the margins [64]. Vergès views violence as a structural element of capitalism and patriarchy and therefore revisits the notion of gender-based violence solely through a female victim/ male perpetrator lens [24]. Vergès argues that there are structural obstacles preventing equality among women and among men, particularly racialized men, or people with non-binary gender identities, or across other forms of vulnerabilities.

A decolonial feminist analysis alerts us to the ways racist rhetorics have constructed certain groups as dangerous while simultaneously rendering invisible or enabling other structures of violence. Take, for example, discourses surrounding sexual violence that invoke remnants of colonial stereotypes related to the hypersexuality of racialized men, emphasizing their cultural identities as inherently violent [65]. In humanitarian contexts, the pathologizing of racialized men has perpetuated the narrative of heightened risks for conflict-related sexual violence. Meger (2016) argues that this has transformed conflict-related sexual violence into a commodity, an object with exchange value that is exploited by various actors, including aid organizations, politicians, and even academics, “as a means to obtain funding and as a currency for influencing political negotiations” (p. 156). This “fetishization” of violence has been used to justify escalating militarized presence and surveillance in the Global South [66].

Decolonial feminist thought is resolute in depatriarchalizing conversations about violence against women and addressing the global context within which violence is naturalized. The preceding example reflects the need to de-centre the female victim/ male perpetrator lens that dominates conversations about violence, allowing for reflections on violence as a structural element of imperialist, racist, and capitalist oppression and exploitation and how these forms of violence mutually reinforce one another. Vergès argues that a decolonial analysis of violence against women and girls cannot be separated from ongoing histories of colonialism and capitalism that have legitimized precarious employment, land theft, destruction of public service, massacre, rape, and have instead disguised them as humanitarian interventions or civilizing missions [2,24]. Decolonial feminism recognizes that the stark inequality between the Global North and South that persists is based on the exploitation of the Global South’s resources and that racialized women bear the burden of discrimination, relentless exploitation and degradation of ecosystems. Razack observes that culturalizing violence against women often leads development program actors to adopt a white saviour role and transform women in the Global South into objects to be saved, often by Western women [61]. She argues that unless programs are framed within strong anti-racist and anti-capitalist approaches that provoke reflections on deeply ingrained assumptions in development work, they are unlikely to tackle the issue of violence effectively.

#### 3.2.1 An interrogation of culture.

A decolonial feminist perspective is cognizant of the epistemological depiction of regressive environments for women and girls in the Global South. Non-Western cultures, particularly of the Global South, are stereotyped as primitive and uncivilized, especially as it relates to gender equality. Decolonial feminists warn against such simplistic and negative attribution to non-Western cultures, citing the colonial and imperialist contexts within which the idea of culture came about.

Abu-Lughod’s feminist critique and interrogation of the concept of culture reveal how the concept has long been used as an essential tool for making the “other” [67]. Cultural differences that defined the way people lived were used as justification for colonization and the continuous imposition of interventions to civilize communities. In the same vein, Ahmed [68] critiques Western feminism’s complicity in what she calls “colonial feminism,” which is constantly seeking to undermine and devalue local cultures by presuming that the only path to emancipating women is through the adoption of Western models. Lazreg [69] argues that this has made feminists from the Global South feel under pressure to choose between their feminisms and their culture. They often find themselves in a position of either defending their cultural practices against misrepresentations by feminists or taking pride in discussing those practices that may be viewed negatively yet are sensational. This tension typically reinforces the dominance, legitimacy, and perceived superiority of Western feminism [69].

In discussing culture, we take as a starting point Abu-Lughod [67]’s critique of the problematic history of the study of culture, which created a hierarchical divide between the knowledgeable scholar (the self) and the person whose culture is being studied (the inferior other). Such approaches continue to reproduce hegemonic narratives of defective lifestyles, particularly in the Global South. Bradley [70] argues that the word “culture” often raises more problems than it solves, given that its foundations were rooted in scientific laws that viewed human existence as a continuum from savagery to barbarism and civilization. Any interrogation of culture must confront the power relationships inherent in self-other separations. Interrogations must also acknowledge that the dividing lens through which culture has been studied is a fundamental method of enforcing inequality.

Anthropologists widely understand culture as an invisible lens through which people learn to view and interact with the world and shape their beliefs, behaviour and morals [70]. Often acquired by one’s membership in a social group, culture is manifested at different layers of depth and encompasses visible and invisible traits, including value systems and basic assumptions that guide behaviours. For example, Ghanaian Philosopher Kwasi Wiredu describes the vast nature of culture in African contexts, which underlies their lifestyles, institutions, worship, courtship, nature exploration, clothing, warfare, and peacekeeping [71]. Culture also influences their organization of systems, education, and justice. He provides examples of African culture, including kindness to strangers, reverence for ancestors, belief in good and bad spirits, the notion of one supreme God, high esteem for large families, higher esteems placed on group efforts, obedience to authority, reconciliation capacity, informal child education, ideas of predestination, reliance on oral traditions, and the importance of elaborate rituals and ceremonies throughout life stages [71]. Consider how, for instance, the emphasis on communal efforts, as described above, would sharply contrast with ideologies for gender equality that reflect a Western worldview that defines the person as independent and separate from others, with goals that pertain solely to the self.

#### 3.2.2 Gender equality as cultural erasure.

A decolonial consciousness brings to the fore contestations about Western conceptions of what is good, moral, or just, which have led to the misrepresentation of cultures from the Global South. Sticking with the African example, it is often argued that feminism is un-African; a sentiment suggesting that feminist action is a Western concept and that African cultures are devoid of gender justice [19,60]. Approaches to gender equality tend to begin with undoing and erasing culture and adopting Western assertions of being, thereby validating and sustaining saviourism narratives from the Global North. A decolonial feminist lens forces us to critically examine colonial patriarchal constructions of girl/womanhood that have survived in contemporary times and de-link from Eurocentric and imperialist worldviews and epistemologies. Maria Lugones draws on Oyèrónkè Oyewùmí and Paula Gunn Allen in her assertions that gender stratification, as we practice today, is a colonial construct [16]. Assumptions of male superiority, even in contemporary African contexts, result from the racialized and capitalist process of hierarchical gender stratification.

A decolonial consciousness does not seek to romanticize culture but to reclaim its positive attributes by interrogating cultural practices, while challenging the weaponization of culture against social groups who live at the margins of society [16,23,60]. A decolonial feminist lens critiques colonial erasures of women’s bases of power and the abuse of culture to consolidate power in men’s hands. Weaponizing culture in this way was key to the colonial disruption of the sociopolitical process of many communities and establishing what Tamale [19] calls the capitalist patriarchy. Men dominated the public market sphere, and women, who had previously held significant power (in settings where this had been the case), including economic power, were relegated to the private domestic sphere. Among other things, such a structure upheld the remuneration of labour in one sphere (public) and the expected gratuitous offering of labour from the other sphere (private). This (re-)structuring was essential for capitalist production to maximize profits in the colonies.

Critiques of cultural history anchored in decolonial and anti-capitalist arguments are important for understanding how societies, especially in the Global South, have grappled with exclusionary facets of colonization. The preceding arguments also demonstrate that culture is, in fact, a “dynamic evolving hybrid of histories and geography” [49]. Abu-Lughod [67] cautions against associating culture with problematic connotations of homogeneity, coherence, and timelessness, which constructs groups of people as fixed entities. These descriptions tend to obscure differences among groups and smooth over contradictions, as well as people’s changing motivations and circumstances. Culture, whether it is conceived as a set of behaviours, customs, or traditions, should not be understood as innate and natural, but as learned, sometimes imposed, and subject to change.

A decolonial understanding of culture serves as a repudiation of hegemonic perspectives of womanhood and girlhood. The Western experience of girlhood is marked by individualism, entrepreneurship, delayed marriage and motherhood. Therefore, non-Western girls who prioritize solidarity over individualism or who value the well-being of family, faith and community are deemed “backwards”. Leila Ahmed views this argument as pertinent in understanding the West’s obsession with unveiling women and the fixation on the veil as a sign of domination [68]. She describes the veil as a symbolic marker of cultural identity and women’s status in the Muslim world. Veiling can contribute to girls and women’s experience of freedom of movement in public, easier work relations in mixed-sex settings, respectability in the eyes of their community, greater economy, and social conformity [3,68]. Too often, people’s desires to maintain their cultural identity are met with scorn. It is for this reason that Khoja-Moolji [31] worries that development projects targeting women and girls appear to be another individualizing project, whereby prevailing structures of power and authority that influence their lives are not sufficiently interrogated.

Projects that aim to address challenging cultural practices affecting women and girls suggest that their communities are intolerant, conservative and prisoners of their traditions, all while neglecting the dynamism of culture and the cultural assets that afford women and girls inclusion and social support. Evidence has shown that it is often their local communities that provide the strongest support networks and help drive change in the presence of culturally informed and socially contextualized interventions [31]. Bourdieu’s concept of social capital supports this notion, which he describes as deriving advantages from social networks [72]. He notes that every group has delegates who hold power and are responsible for protecting their members, especially the most vulnerable. These delegates can wield power and act with authority on behalf of the entire group.

#### 3.2.3 Harnessing cultural assets.

Cultural resources are essential for the health and well-being of women and girls. A study in Nigeria highlighted that women’s intricate psychological experiences of female genital mutilation/cutting (FGM/C) were shaped by their beliefs in the cultural ‘gains’ of the practice [73]. While women recognized the severe pain and trauma linked to FGM, many supported it because it enhanced their cultural capital by presenting a culturally esteemed image of a complete woman who has command over her sexuality and is capable of childbirth. Cultural capital refers to valuable cultural assets that aid an individual’s social advancement [74], which is “fundamentally linked to the body and presupposes embodiment” [p244]. Bourdieu explains that this embodied capital is often gained through intergenerational transmission of knowledge, shared norms, and rituals. For these women, undergoing FGM not only shaped their collective identities but also granted them recognition and privilege within their society, thereby enhancing their social acceptance, support, and self-esteem.

Feminist scholars have argued that it is within these cultural knowledge systems that solutions arise, because through these systems, knowledge practices have evolved for generations and have proven flexible enough to cope with change [75]. Tamale views culture as “a site of multiple possibilities where Indigenous groups, as agents, actively and strategically invent and reinvent themselves” [19]. Therefore, approaches to ending FGM/C must first situate it within its cultural context as a perceived cultural asset; only then is it possible to generate alternative strategies and practices to favour women and girls’ gains in cultural capital.

Creating possibilities for transformation means leveraging the social resources of women and girls to facilitate collective action that improves their lives. Evidence from African contexts indicates that young women and girls often feel they lack the power to challenge traditions. In contrast, older women in families, who are respected as bearers of tradition, utilize their established authority to advocate for changes that benefit women and girls [76]. These women play a crucial role in the intergenerational transmission of traditions, as they represent the existing power structures that uphold certain practices. However, they are also open to change and have been key in driving the adoption of improved child health practices, including the discontinuation of FGM/C [77,78]. Chatterjee defends traditional community structures as possessing effective resources, sometimes surpassing those of modern state institutions, to mediate conflicts, embrace diversity, and foster the formation of a more adaptable and inclusive community. He views the ‘little traditions’ of local community life as the result of centuries of adjustment to social change [35].

### 3.3 Work as intrinsically liberating

In interrogating the ideological underpinnings of gender equality programs, our findings indicate that programs capitalize on patriarchy as the root cause of rampant poverty among women and girls. In a report by the NoVo Foundation & Nike Foundation in 2018, the authors insist that women and girls face limited economic opportunities and poverty resulting from gender-based oppression at the family and community levels [45]. They state that “because the problem is gendered, so too must the solution.” Therefore, poverty must be addressed by bridging the gender divide in business opportunities. A report by the Bill & Melinda Gates Foundation in 2014 also explains that gender inequality is the root cause of poverty and inequality, as experienced by women and girls [79]. The program logic for Women’s Strong International shares a similar sentiment in arguing that gender norms are what keep women and girls from enjoying economic freedom [80]. To address the problem with a gender-solution, Nike Foundation remains resolute in enabling girls to gain access to professional experiences and exposure, skill-building and jobs. They situate “girls are economic actors, and position “the private sector as a critical part of the environment that defines and creates economic opportunities for girls, because “when the private sector engages with girls in age-appropriate ways, new doors can open.” (p.66). They argue that development programs have long failed to recognize girls as economic actors whose employment accrues benefits to employees and the girls themselves. Their reasoning appears to be straightforward; a simple solution to poverty can be found in work opportunities for women and girls, especially within the private sector.

This rhetoric, decolonial feminists argue, paints a flawed picture of poverty. The construction of women and girls as key solutions to global poverty relies on depoliticizing poverty in ways that make invisible the power relations that produce inequality and narrows understandings of the world’s wealth divergence [2,3]. In the words of Sweis

Poverty [in NGO efforts] is articulated less as a political-economic concern for governments and more as a pragmatic problem that can then be dealt with by a concerned public and by educating girls, one at a time. Women and girls, as individuated subjects, are constructed as national panaceas, as the answer to global poverty and underdevelopment [81].

Hengeveld concurs that key development interventions gloss over the exploitative practices and overall structural violence that the “free” market inflicts on the Global South and instead focus on cultural, patriarchal and familial practices as the world’s greatest problems [82]. But as Samir Amin points out, it is important to understand that economic development in rich countries of the Global North and underdevelopment in poor countries of the Global South are opposite faces of the same coin [83]. Long histories of systematic exploitation by rich countries who enforced a racialized global hierarchy that weakened the economic power of poor countries contribute to maintaining unequal structures of the world economy and the subsequent persistence of poverty in poor countries of the Global South. Furthering grand narratives of infinite economic progress as obtainable in the Global North inadvertently defends these extractivist economies, which, as we have discussed, were historically founded on racial and capitalist exploitation of people and resources of the Global South [5].

A decolonial feminist analysis scrutinizes the way asymmetrical power relations inherent in the global capitalist system are etched into women’s bodies. NGOs, such as Women Strong International, insist that the entrepreneurial spirit of the economically powerless women and girls is what promises economic prosperity of a nation [84]. Programs fully embrace the neoliberal logic that when equipped with the right education and entrepreneurial skills, women and girls can solve global economic problems by pursuing their own economic and social development, all while forgoing any accountability to the state. Entrenched in the belief that market forces lead to the best economic outcomes is the idea that work is intrinsically liberating [82]. Programs point out that since women’s work within the home is economically undervalued by their families, access to jobs is the only pathway out of poverty, which also guarantees women and girls the ability to make informed choices and effectively exercise their voice and agency [59]. The relationship between work and the emancipation of women and girls’ bodies from the “traditional” is not accidental. It purposefully furthers the motives, techniques and interests of wealthy corporations who are lauded for their dedication to the poor and marginalized women and girls of the Global South, but have, to a large extent, remained wealthy by legitimizing inequality and exploitation. Quite evidently, and most relevant to this paper, are corporations that rely largely on the cheap labour of women and girls [3,82].

Contrary to the corporate logic of NGO programs, the literature has long disproved that work in and of itself alleviates poverty. A recent ILO report indicates that while the world celebrates historically low levels of unemployment, especially for women, millions are trapped in cycles of economic marginalization. In sub-Saharan Africa where 86.6% of people employed in 2024 were in informal sectors, millions of them faced extreme forms of working poverty because informal sectors often lack social protection, labour rights and regular liveable incomes; therefore, while people were employed, they were not in productive and decent employment and still lived in poverty [85]. These forms of employment result from global structural injustices, such as weak labour laws, from which multinational corporations typically benefit.

Take, for example, the various sweatshop scandals that highlight how companies such as NIKE, an influential authority in gender and development through its NGO, the Nike Foundation, perpetuate and profit from the exploitation of girls and women [1,7,24]. Women and girls, who often comprise the largest segment of the sweatshop workforce, decried the lack of decent work protections and living wages, among other issues. Similar literature has demonstrated adverse health outcomes such as respiratory and cardiovascular illnesses, musculoskeletal illnesses, and mental health issues for the sweatshop workforce [86]. It is reasonable to assume that working in these environments is far from liberatory or emancipatory. The ILO report argues that for employment to fulfill a proper role in lifting people out of poverty, it has to be of good quality (decent work). As Hengeveld argues, the power asymmetries inherent in the global capitalist system “cannot be entrepreneured away no matter how capable women and girls are” [82]. Therefore, when programmes fail to address decent work and income security but instead focus the blame on patriarchy while expecting women and girls to fix global socio-economic ills, their efforts towards poverty reduction remain incomplete and possibly futile.

While the neoliberal assumption of market participation as inherently liberatory is problematic, decolonial work has also acknowledged that women’s expression of agency within paid work involves complex and situated forms of agency. For example, Desai demonstrates how class influences the valuation and governance of Gujarati women’s bodies, labour, and health [87]. In a class-based health system, lower-income women and their uteruses are often deemed ‘disposable’ compared to those of higher social classes. This stratification limits some to a constrained, yet pragmatic decision to choose a hysterectomy as a strategy to protect their precarious livelihoods and the identities linked to their work. This highlights how women’s agentic acts of survival unfold in response to an exploitative gendered labour economy and are structured by enduring hierarchies of class and power. Yet, for Wilson, recognizing this and other expressions of agency within exploitation must not obscure ideologies and conditions that necessitate such strategies in the first place [88].

### 3.4 Universal discourses in local settings

As evident from our findings, NGOs operate on the basis of human rights, underpinned by universal frameworks. Most programs apply human rights-based language in their information, education and communication materials. Many aim to advance gender equality through advocacy for a human rights agenda. Let Girls Lead explains that “a human rights framework is key to successful advocacy efforts for and with girls, as it provides tools for analyzing the root causes of the problems and inequities that girls face” [58].

Contemporary feminist analyses of human rights, including laws, institutions, and practices, argue that it has historically been “male-dominated, male-identified, and male-centred” [89]. Its liberal ideological foundations, theorized in the context of elite White male experiences, excluded the experiences of women or marginalized groups. Furthering this conversation, feminists from the Global South have highlighted the tensions in deploying the Western perspective of human rights as a universal discourse applicable to all human beings in the same way, regardless of geographical location and cultural context [90–92]. They contend that it is impossible to speak about universal human rights because of the inequities and contradictions inherent in the human rights system. Take, for example, the colonial imprint in human rights instruments such as the Universal Declaration of Human Rights (UDHR), whose discourse has persisted alongside colonial systems since its inception in the mid-20th century [19]. The UDHR which serves as a significant framework for NGOs promoting core human ideals, was created with the involvement of both colonizers and those who upheld institutional apartheid and racism at that time; members and representatives from the Global South were notably absent from conceptions of universal human rights [19]. This paradox gives but a glimpse into the inconsistencies, contradictions, and colonial legacy embedded in the human rights system.

Abu-Lughod points to the hegemony of human rights language in advocacy for gender equality and its commercialization or association with the corporate world [93]. Their interpretations and narratives, steeped in Western philosophical values, emphasize the autonomous individual, with social individuality taking precedence over collective norms. They valorize neoliberal assumptions of personhood and success. Discussions about human rights in programs often portray “culture” and “rights” as conflicting concepts. For example, Save the Children shares that “Local Customary Laws are discriminatory (e.g., Customary Order allows each ethnic group to follow and make decisions based on their customs and traditions. In El Salvador the legal framework (the Family Code) allows girls to be married if parents/caregivers approve the union if a girl is pregnant or has children), therefore... implementing these global Guarantees to Girls’ rights can help empower girls.” These programs perceive women’s and girls’ rights as inherently in tension and fundamentally at odds with their cultural values [19]. However, ongoing decolonial efforts are shifting understandings of culture and its amenability to change, suggesting that rigid interpretations of culture stem from a colonial conception of culture as being inimical to reform [4,67].

Moreover, various programs prioritize formal legal and law enforcement structures to guarantee and protect the health rights of women and girls, often overlooking non-state authorities like chiefs or councils of elders. For example, programs such as UNFPA-UNICEF and WomenStrong International envision the protection of women and girls’ rights to health exclusively through formal legal avenues involving police, prosecutors, and judges, positioning them as the most effective means to safeguard women’s health and rights [57,94].

This interpretation excludes protection from other legal systems. At this point, it is essential to recognize that legal pluralism, characterized by the overlap and coexistence of various legal systems within a particular socio-political context, is prevalent in many countries of the Global South [95,96]. This typically involves the co-existence of formal legal systems, which depend on the state for law enforcement, alongside people-centric or community-oriented non-state legal systems. Decolonial feminists remind us that colonial interactions shaped legal frameworks in many countries of the Global South [19]. These systems reflect colonial histories of domination through interlocking systems of capitalist and patriarchal oppression. These systems, as decolonial feminists point out, were alien, adversarial, and non-participatory in the regions where they were imposed. The ideological justifications for these systems in the first place were the absence of “proper law” and the labelling of legal practices and institutions in colonized societies as pathological deviations from proper laws.

The deference to these legal structures as the sole protectors of women’s rights positions them as non-gendered or non-racial, masking how these formal legal systems contribute to or are complicit in institutionalizing racialized and gender hierarchies [24,89]. But as evidence shows, increased regulation and laws have not made a significant impact in ensuring the health rights of women and girls [56,97]. This reifies various arguments by decolonial scholars that while it is widely recognized that the formal legal system is only marginal to the experience of day-to-day justice in these regions, lasting effects of coloniality continue to position them at the top of the legal hierarchy, overshadowing other forms of law [19,95].

Evidently, the moral crusade of international NGO programs aimed at saving girls in the Global South and ensuring their human rights makes assumptions that childhood in the Global South is fundamentally wrong [98]. For example, in communities built on the bedrock of interdependence and compassion, care work serves as a key feature and determinant of childhood, helping young children develop responsibility and cooperative behaviour [99]. However, interventions targeting children often view care work merely as a burden and a deficit and go so far as to claim that children are “despised” or “captives” if they do not adhere to Eurocentric ideals of childhood. Many decolonial scholars have countered such Eurocentric arrogance and racist distortions by arguing that human rights discussions existed in the Global South prior to colonization. They argue that these discussions have featured distinct perspectives on rights, order, and justice and have always been integral to the social and political ethos of these regions [19,90–92,100,101].

On the issue of care work, decolonial feminists argue that the focus on the individual child in universal human rights charters is, in and of itself, an inherent conceptual bias of these charters. Other conceptions of human rights view children not as atomized individuals but as members of social groups, thus integral to collective family endeavours [101,102]. These charters emphasize the relational aspect of care work and the connections between rights and duties. The African Charter on Children’s Rights, which reflects a conception of childhood and children’s rights within African contexts, articulates not only the responsibilities of the family, society, and the state towards children but also acknowledges the duties of children to help preserve solidarity and harmony within the family [103]. A study in Ethiopia demonstrated that children perceive care activities as essential for shaping family life and recognize the associated social benefits. They expressed agency through unpaid care work in order to benefit themselves directly or indirectly [104]. Understanding the context of care norms and their significance in communities can reveal strategies to reduce and redistribute the inequitable gendered division of unpaid care, rather than viewing childhood care work merely as a deficiency.

Unveiling the Eurocentric emergence of human rights discourses weakens the legitimacy of claims to their universality and invites contextualizing these concepts. To borrow from Tamale & Oloka-Onyango (1995), “understandings of rights should be sought through intra-cultural and cross-cultural dialogue, rather than as an abstract, culturally neutral proposition which it can never be. Without this dialogue, any international action is doomed to be dominated by a single ideology or perspective.” [91]. Spivak raises this issue in a critical examination of the human rights discourse, highlighting the epistemic gap between human rights advocates and those they aim to protect in the South [105]. Establishing a universal concept of human rights arrogates to so-called experts the position of dispensers of these rights, justifying ongoing supervision and intrusion into the lives of the subaltern. In response to this, Spivak advocates for the recognition of diverse knowledge systems, decision-making strategies, and governance structures.

While challenging the assumptions underlying the dichotomy between rights and culture, we must recognize the misappropriation of culture, particularly by those with political and socioeconomic power who perpetuate essentialized versions of customs in the Global South and abuse rights under the guise of culture. The decolonial feminist struggle resists the weaponization of culture as a means of maintaining inequities at various levels of oppression, including gender, class, and sexuality. Instead, it calls for examining and contesting cultural practices that have been weaponized against social groups operating at the margins of society. Decolonial research can pave the way forward by valuing and reclaiming indigenous voices and knowledge forms. The specific experiences of women and girls from the Global South, their perceptions, and the complex subjectivities they embody, need to be foregrounded in knowledge production.

Furthermore, while we pay attention to the unequal geopolitical relations that constitute the global rights framework whereby local actors are considered adopters rather than necessarily the producers of these rights frameworks, Anthropological scholarship invites us to make sense of its travels and translations across forms, offering an opportunity to discuss the epistemic infrastructures of the human rights discourse [93,106]. Taking an actor-centred approach, the global human rights discourse can be understood as a site of epistemological struggle where local actors, such as women, negotiate, translate and unsettle the colonial imposition of universality [107]. Levitt & Merry’s [108] description of ‘vernacularization’ explains that, rather than a linear transfer of rights from the global to the local, human rights ideas interact with local knowledge systems, where they are influenced by specific histories and power dynamics and integrated into local interpretations, while also meeting resistance to their adoption in its entirety. This centres Global South women not as passive recipients of these Western-generated norms but as agents actively resisting, negotiating and transforming the imposition of universality.

## 4 Conclusion

In conclusion, while closing gender disparities is important, the meaning attached to gender is central to the struggle and requires unpacking because its construction is wrought through colonial epistemologies and practices [19,23,44,109]. Decolonial feminists prioritize indigenous identities, particularly within racial/gendered feminist contexts, by recognizing the conceptualizations of gender that pre-existed European modern/colonial gender systems. They contest the universalization of gender categories, which serves as a fundamental organizing principle across all societies throughout history. For example, Oyěwùmí argues that in Yorùbá societies of West Africa, the introduction of gender concepts led to a clear classification of women as subordinate to men in every context [23]. Lugones theorizes that colonization established notions of race and gender; the enforcement of race accompanied the inferiorization of racialized people, and the imposition of gender accompanied the inferiorization of racialized women [109].

Current understandings of gender have been preserved through epistemological control and domination, or, as Quijano explains, through the coloniality of power [109,110]. The coloniality of power, particularly through knowledge control, focuses on the intellectual dominance exerted by Western-centric knowledge production and distribution practices. This dominance, achieved through colonization, is maintained through ongoing intellectual and cultural imperialism that continuously marginalizes alternative knowledge systems and ways of organizing [51]. The coloniality of knowledge served as a means of control to detach colonized communities from their capacity for autonomous thought. This dynamic is still evident today in the relationships among institutions, academics, and systems of knowledge between the Global North and other parts of the world [42,43]. International NGO’s practices are as they are in part because the West remains at the centre of producing and disseminating knowledge, while the rest of the world consumes this knowledge. Lugones insists that coloniality is maintained in feminist studies by implementing Western understandings of gender and erasing the various conceptualizations of gender that pre-existed European modern/colonial gender systems [22,111]. The coloniality of gender is deliberate as it facilitates the control over sex, subjectivity, authority and labour.

Our analysis challenges the belief that a Western framework for analyzing society and social dynamics can be universally applied. However, the decolonial project does not aim to return to a romanticized pre-colonial past. Instead, it seeks to recognize how colonialism has undermined the bases of power occupied by women and girls and established boundaries that diminish and hierarchize their positions as knowledge holders. Modern discourses about the liberation of women and girls in parts of the Global South continue to uphold the power dynamics established during colonial times. Disele-Pitso and Tamale recommend a delinking from Western narratives in order to relink and affirm modes of existence [19,60]. De-linking “brings to the foreground other epistemologies, other principles of knowledge and understanding, and consequently, other economy, other politics, other ethics” (Mignolo, 2007, p.453) [19,112,113].

In the spirit of encouraging a delinking from Western narratives as recommended by Pisto and Tamale [19,60] and in light of the perception of international NGOs as the preeminent organizational form capable of implementing global commitments within local communities, in this paper, we adopted a decolonial feminist perspective to analyze the political economy of gender equality programs by those NGOS and their contradictory, universalizing, and essentialist discourses. Through a document review of international NGO programs targeting women and girls, interpreted using a decolonial feminist perspective, we investigated contemporary framings of gender equality in the Global South. The findings show how gender equality rhetoric is rooted in neoliberal framings and establishes links between NGO discourses and their coloniality of knowledge. The findings suggest that many gender equality initiatives are hosts to oppressive forces that derail gender equality efforts. To that end, we emphasize the importance of integrating a decolonial feminist perspective into gender equality programs to uncover and dislodge the myriad manifestations of colonial influences. Failing to do so, international NGO programs and policies will either be counterproductive or limited to partial and temporary success.

However, analyzing documents has certain limitations. While organizational program or intervention documents provide useful insights into how organizations communicate their priorities, assumptions, and expected outcomes, they do not offer a complete picture. These documents often present curated narratives that may not fully capture the complexities of different perspectives or practices [114]. We recognize that this approach lacks contextual information, and by excluding non-written communication, there is a risk of misinterpreting meaning. To mitigate these issues, we have clarified our document selection and analysis process by employing the READ framework and by practicing reflexive interpretation.

### 4.1 Partial alignments and bright spots

While much of the analysis reveals the reproduction of colonial logics in gender and development programming, several documents also demonstrate partial alignments with decolonial praxis. For instance, Strong Girls Make Strong Women: A Handbook for Girls’ Clubs emphasizes localized, adaptable content and the ICRW incorporates elements of participatory design and youth-led monitoring that foreground local agency. These initiatives, though still situated within broader donor logics, attempt to centre girls and women’s voices in defining program priorities and success indicators. These examples provide insights into relational accountability and the path towards equity-driven knowledge production [115]. Aligned with Vergès’s perspective on a decolonial transformation, these examples suggest that such a process is not a straightforward or linear “victory” but an ongoing, contested journey that respects the complexity of the struggle [5]. It acknowledges the partial wins and ongoing forms of resistance within limited structures. However, a decolonial feminism remains committed not only to reforming existing systems but also to challenging all forms of oppression [5]. Still, considering these examples alongside harmful repetitions promotes a more nuanced understanding of the field and highlights both the persistent colonial hierarchies and the rise of more equitable, collaboratively developed forms of knowledge and action.

### 4.2 Turning the analytical lens towards practice

As we move towards structural change, we explore how decolonial feminist theory can inform and shape practice. Aligned with the Global Health Decolonial Movement in Africa’s call for “common sense” approaches, we discuss strategies for challenging entrenched colonial hierarchies and racism in development practice. By reimagining the design, governance, measurement practices, and financing of development initiatives, we present the following recommendations as a starting point to foster reflexive, relational, and contextually grounded dialogue, practice, and policy on gender equality programming.

**Design** principles with a decolonial feminist orientation will begin with women, girls and people of diverse gender identities defining their own health priorities beyond donor agendas or externally imposed program logics. The field of global health and development has long been dominated by those with disproportionate power, who generate knowledge and interpret health experiences and aspirations of less powerful groups, reproducing epistemic injustices and silencing alternate ways of knowing [116]. A decolonial feminist approach will elevate Indigenous, local, and experiential knowledge systems as legitimate evidence and embedded intersectional and anti-oppressive lenses that encompass how gender is mediated through colonialism, patriarchy and capitalism while recognizing the intersections of race, caste, class, age, disability and sexuality. In practice, this could entail co-creating tools and processes of meaningful dialogue with diverse collectives, including feminists, women, LGBTIQ + , Indigenous, and Afro-descendant communities. In the program curriculum, it is important that they reflect on the legacy of former colonial relationships and how they continue to impact global health and development practice [117].

**Core governance** within a decolonial feminist framework promotes shared power, collective decision-making, and accountability to the communities involved. It rejects viewing communities through a deficit lens as passive beneficiaries and instead highlights their inherent strengths, assets and epistemic authority. For instance, leadership structures in community work should acknowledge and include multiple and context-specific forms of authorities who influence women’s and girls’ lives and can serve as allies in transformative change [77,78]. Equally important, community engagement must be rooted in reciprocity, rejecting the northern-centric extraction of data, ensuring that communities retain agency over how information is used, shared and translated into action. Accountability towards the community must be prioritized rather than upwards towards donors or the state, ensuring that those most affected by inequalities are best positioned to shape governance and ethics of interventions [117].

**Measurement** choices with a decolonial feminist approach would begin with a feminist theory of change, one that reimagines health and gender beyond biomedical or Western binary systems. This approach will ensure that measurement moves beyond hegemonic and technocratic perspectives portrayed as “natural standards” and focuses instead on relational accountability [118] Indicators must be context-specific and power-sensitive, acknowledging how hierarchies within systems influence trust, participation, and meaning-making in health programs. For example, measurements can include relational indicators such as trust in systems, collective power, perceived equity in knowledge exchange between community members and INGO staff [117,119]. By grounding evaluation in lived realities and community-defined priorities, measurement can become a political act of epistemic equity [115].

**Financing** from a decolonial feminist perspective views money as inherently political, serving as a tool that either reproduces or dismantles inequality. In a report by Oji Oti et al., [120] the authors note that a decolonial feminist reimagining of philanthropy aims to redirect and redistribute wealth, not as charity but as an acknowledgment that colonial and patriarchal systems have often enabled unchecked wealth accumulation at the expense of marginalized communities. A decolonial feminist approach replaces short-term, project-based grants with longer-term funding that emphasizes holistic support through programs. These approaches, grounded in mutual trust and accountability and guided by intersectionality, challenge patriarchal and colonial control over funding, such as hierarchical oversight by Global North donors, and instead empower those closest to the issues, like women, Indigenous peoples, feminist and gender-diverse groups, and other grassroots collectives, to define their own priorities.

## Supporting information

S1 Table Code book and selected supporting quotes from documents. (XLSX)
